# Ultra-sensitive heterodyne detection at room temperature in the atmospheric windows

**DOI:** 10.1515/nanoph-2023-0787

**Published:** 2024-01-18

**Authors:** Mohammadreza Saemian, Livia Del Balzo, Djamal Gacemi, Yanko Todorov, Etienne Rodriguez, Olivier Lopez, Benoit Darquié, Lianhe Li, Alexander Giles Davies, Edmund Linfield, Angela Vasanelli, Carlo Sirtori

**Affiliations:** Laboratoire de Physique de l’École Normale Supérieure, ENS, Université PSL, CNRS, Sorbonne Université, Université Paris Cité, 75005 Paris, France; Laboratoire de Physique des Lasers, CNRS, Université Sorbonne Paris Nord, 93430 Villetaneuse, France; School of Electronics and Electrical Engineering, University of Leeds, Woodhouse Lane, Leeds LS2 9JT, UK

**Keywords:** infrared detection, unipolar quantum devices, frequency stabilization

## Abstract

We report room temperature heterodyne detection of a quantum cascade laser beaten with a local oscillator on a unipolar quantum photodetector in two different atmospheric windows, at 4.8 µm and 9 µm. A noise equivalent power of few pW is measured by employing an active stabilization technique in which the local oscillator and the signal are locked in phase. The measured heterodyne noise equivalent power is six orders of magnitude lower than that obtained with direct detection.

## 1 Introduction

Promoting uncooled sensitive and high bandwidth photodetection in the mid (3 μm < *λ* < 8 μm) and long (8 μm < *λ* < 15 μm) wave infrared range is a challenging problem essential for several applications, spanning from high-bit-rate free-space communications in the atmospheric windows [1] to quantum metrology [2], [3]. In addition, a great research effort has been dedicated into LiDAR systems, in particular for automotive applications [4] and for the simultaneous measurement of velocity and distance of molecules in the atmospheric windows [5]. For the development of LiDAR technology in the wavelength ranges considered here, the detection of very weak signals is a major challenge. Heterodyne detection, where a weak signal is coherently mixed with a powerful local oscillator on a fast detector, is a promising technique to address this issue. We propose and demonstrate the implementation of this technique in the thermal infrared range by using unipolar quantum optoelectronic devices, such as quantum cascade lasers (QCLs) [6], quantum well infrared photodetectors (QWIPs) [7] and quantum cascade detectors (QCDs) [8]. Indeed, QCLs feature today continuous-wave single-mode emission with output powers over 100 mW and are, therefore, powerful sources of coherent radiation that can be used as local oscillators [9], [10], [11], [12]. On the other hand, QWIPs and QCDs show a high optical saturation power [13] and large frequency bandwidth, exceeding 100 GHz [14], making them ideal heterodyne receivers. The performances of such detectors in terms of signal-to-noise ratio, frequency bandwidth and temperature operation have been recently improved by inserting them into metamaterial architectures that insure a good coupling with free space radiation and reduce the electrical area [15], [16], [17]. Additionally, unipolar quantum optoelectronic devices profit of the very mature semiconductor packaging technology that makes them perfect chips for compact instruments and optical systems. The sensitivity of the heterodyne detection depends on the stability of the beatnote between the signal and the local oscillator, because the signal to noise ratio is directly proportional to the integration time. When free-running QCLs are used in heterodyne systems, the noise equivalent power (NEP) is set by the different sources of noise limiting their frequency stability. Typical free running linewidths are of few MHz due to the noise arising from electric current [18], [19] and other low frequency fluctuations that set the value of the noise equivalent power (NEP). In this work, we mitigate the effect of these fluctuations thanks to an active stabilization technique in which the local oscillator and the signal are locked in frequency and phase by the injection of a correction current derived from a phase-lock-loop (PLL) [2], [20]. As a result, we measure a noise equivalent power as low as few pW, more than one order of magnitude lower than previous demonstrations [16]. The paper is organized as follows. After presenting our experimental set-up in Section 2, we discuss in Section 3 the sensitivity of optical heterodyne detection with free-running QCLs by measuring the associated NEP. Then, in Section 4, we focus on the effect of the stabilization technique on the reduction of the NEP. Finally, in Section 5, we discuss possible ways to further improve our system.

## 2 Experimental setup

**Figure 1:**
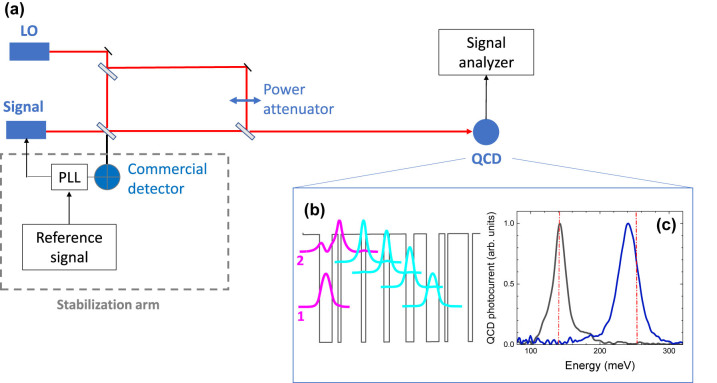
Heterodyne detection experiment. (a) Diagram of the experimental setup. (b) Conduction band profile and square moduli of the relevant wavefunctions of one period of the 4.8 µm QCD used in the experiment. (c) Room temperature photocurrent spectra of the two QCDs. The dashed red lines indicate the laser emission energies. LO, local oscillator; PLL, phase-lock loop; QCD, quantum cascade detector.

The experimental setup for optical heterodyne detection is presented in Figure 1(a). The heterodyne beatnote is produced by two QCLs, the first is the local oscillator (LO), the second is referred as signal. Two different set-ups have been realized, operating in two different atmospheric windows, at 4.8 μm and 9 μm. Each set-up employs a couple of commercial distributed feedback (DFB) QCLs operating in continuous wave at room temperature (from Ad Tech Optics at *λ* = 4.8 µm and from Thorlabs at *λ* = 9 µm). Two different optical paths have been defined for the QCL beams. The first arm, delimited by a dashed line in Figure 1(a), is devoted to the active stabilization between the two QCLs by measuring the heterodyne beatnote on a commercial HgCdTe photodetector (Vigo Photonics) and then comparing it to a radiofrequency (RF) stable reference signal (Rohde & Schwarz SMC100A). A home-made PLL is used to actively control the current injected in the signal laser in order to phase-lock the latter to the LO. This results in a significantly narrowed beatnote signal down to the sub-Hz level [20]. The second arm of the setup in Figure 1(a) is used for the coherent detection of the signal by measuring the heterodyne beatnote with the LO on a QCD. QCDs [8], [21] are unipolar infrared photodetectors that can operate in photovoltaic mode at room temperature. The active region is based on the periodic repetition of tunnel coupled quantum wells. Figure 1(b) represents one period of the QCD: mid-infrared photons are absorbed through an intraband transition between two confined states, labelled 1 and 2, of two tunnel coupled quantum wells [22]. The corresponding absorption spectrum is centred at the transition energy *E*_12_ = *E*_2_ − *E*_1_. Photoexcited electrons relax, by scattering with longitudinal optical phonons, towards the ground state of the following period of the cascade. The QCD operates in photovoltaic mode due to the asymmetry of the cascade region that acts as a pseudo-electric field driving the electrons in one direction only, thus giving rise to a photocurrent. Figure 1(c) shows the room temperature photocurrent spectra of the two QCDs used in our experiments. The detector used in the 4.8 µm set-up is a GaInAs/AlInAs QCD based on a diagonal design (Figure 1(b)), centred at 247 meV (5 µm), while the 9 µm detector, centred at 141 meV, is realized with a GaAs/AlGaAs heterostructure (the same device has been used in ref. [1]). The red dashed lines in Figure 1(c) indicate the emission energy of the QCLs used in our experiments, which are very close to the QCD photocurrent peaks. In order to operate at high frequency, the QCDs have been processed into 50 µm × 50 µm mesa structures that are electrically connected to a 50 Ω coplanar waveguide through an air-bridge for a low-inductance top contact. The device is then fixed on a custom-made sample-holder and wire-bonded onto a 50 Ω impedance matched coplanar waveguide. The room temperature characteristics of the QCDs are summarized in Table 1. More details on the device characterizations are provided in the supplementary material section.

**Table 1:** Main characteristics of the quantum cascade detectors used in the experiment.

	Material system	Number of periods	Photocurrent peak (meV)	Responsivity R (mA/W)	3-dB cut-off frequency (GHz)	*R*_d_ (Ω)
4.8 µm QCD	GaInAs/AlInAs	12	247	1.9	2	1800
9 µm QCD	GaAs/Al_0.35_Ga_0.65_As	12	141	4.2	3	180

## 3 Optical heterodyne detection with free running QCLs

**Figure 2:**
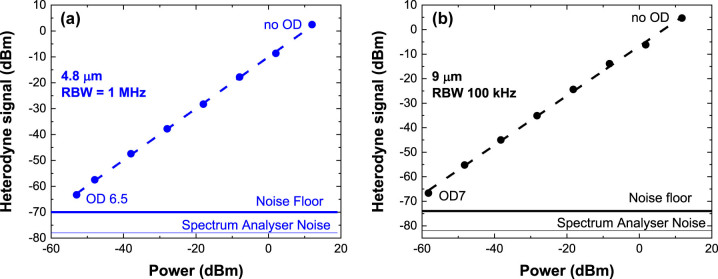
Measured heterodyne power at 4.8 µm (panel (a)) and 9 µm (panel (b)) as a function of the signal power after attenuation by optical densities. The resolution bandwidths (RBW) are indicated in the figures. The frequency of the heterodyne signal is 125 MHz at 4.8 µm and 130 MHz at 9 µm. The QCDs are operated at room temperature in photovoltaic mode. The QCLs are free-running. The heterodyne current is amplified with a transimpedance amplifier. The dashed lines present linear fits to the data, allowing extraction of the heterodyne efficiencies, while the continuous lines indicate the noise floors. OD: optical density.

Before studying the influence of the active stabilization on the coherent detection setup, we have characterized the heterodyne noise equivalent power with free-running QCLs. The stabilization arm indicated with dashed lines in Figure 1(a) is not used for this experiment. The heterodyne power is read on a radiofrequency spectrum analyser (Agilent E4407B) for a constant local oscillator optical power (*P*_LO_ = 45 mW), while progressively decreasing the signal power, *P*_S_, impinging on the detector by inserting optical density filters in the signal path. The heterodyne signal at ∼130 MHz is amplified with a low noise transimpedance amplifier with a gain *G*_trans_ = 5 × 10^3^ V/A. Figure 2 presents the heterodyne power as a function of the signal power at 4.8 µm (panel (a)) and 9 µm (panel (b)), expressed in dBm. A linear behaviour is observed at both wavelengths over more than six orders of magnitude. In fact, starting from a signal power of 15 mW without optical densities, the minimum detectable signal power was 5 nW at 4.8 µm and 1.5 nW at 9 µm. The heterodyne current can be expressed as a function of the signal power as:

(1) $$\left|I_{\text{het}}\right|=2R\eta \sqrt{P_{\text{LO}}P_{\text{S}}}$$

where R is the QCD responsivity (given in Table 1) and *η* is the heterodyne efficiency. The last parameter is an indicator of the quality of the optical setup, as it reflects the matching between signal and local oscillator in both amplitude and phase spatial distribution. The heterodyne power can be obtained from Eq. (1) by accounting for the transimpedance gain (*V*_het_ = *I*_het_ × *G*_trans_) and the instrument input load *R*_L_ = 50 Ω:

(2) $$P_{\text{het}}=\frac{V_{\text{het}}^{2}}{R_{\text{L}}}=\frac{4R^{2}P_{\text{LO}}\eta^{2}{G_{\text{trans}}}^{2}}{R_{\text{L}}}P_{\text{S}}$$

The dashed lines in Figure 2 show the linear fit of the data (in logarithmic scale) by using Eq. (2). A slope of 0.997 ± 0.008 is found for the experiment at 4.8 µm and 1.01 ± 0.01 for the experiment at 9 µm. From the value of the intercept of the linear fit, we can extract the heterodyne efficiencies, which are found to be 55 % at 4.8 µm and 36 % at 9 µm. In Figure 2, the measured noise level is indicated by a continuous line and the resolution bandwidth (RBW) is also provided. Identifying the origin of the noise is essential for a better understanding of the limiting factors of the heterodyne detection setup and possible improvements in the measurements. As they operate in photovoltaic mode, QCDs exhibit negligible dark current noise, which will not be considered in our estimations. Instead, we focus on thermal noise and generation – recombination noise [23], [24]. Thermal noise depends on the detector temperature, *T*, resistance *R*_d_ and resolution bandwidth Δ*f*:

(3) $${i^{2}}_{\text{th}}=\frac{4k_{\text{B}}T\Delta f}{R_{\text{d}}}$$

The calculated values of thermal noise of the detector at room temperature are presented in Table 2. We also report the calculated values of the generation – recombination noise, obtained as

$${i^{2}}_{\text{ph}}=4egI_{\text{ph}}\Delta f$$

with *g* representing the photoconductive gain, considered equal to the inverse number of periods in the absorbing region, and *I*_ph_ denoting the photocurrent (for very low signal powers, *I*_ph_ ≃ *I*_LO_). The values presented in Table 2 reveal that the sensitivity of our experiment is mainly set by the instrument noise floor. The NEP of the system, defined as the value of the power at which the signal to noise ratio is equal to 1, is obtained by linear extrapolation of the data till the noise level. We obtain 1 nW at 4.8 µm and 200 pW at 9 µm. In order to reduce the noise level and improve the sensitivity of the detection, we have used an active stabilization technique to reduce the resolution bandwidth to 1 Hz. As it will be seen in the following, the increase of the integration time induced by the stabilization also allows the measurement of the photocurrent without the need of an amplification stage.

**Table 2:** Different noise contributions for 4.8 µm and 9 µm QCDs in the case of free running QCLs. The calculation includes the transimpedance amplifier gain.

	RBW (MHz)	Calculated thermal noise (dBm)	Calculated generation – recombination noise (dBm)	Spectrum analyser noise (dBm)
4.8 μm QCD	1	−83.5	−86.4	−78
9 μm QCD	0.1	−83.5	−93	−82

## 4 Optical heterodyne detection with stabilised QCLs

The sensitivity of the heterodyne setup is limited by the stability of the beating, which sets the resolution bandwidth and consequently the integration time. Standard ways to reduce laser frequency fluctuations involve passive (i.e. minimizing temperature fluctuations, reducing the noise sources in the setup) or active stabilization of the QCLs. Several active stabilization experiments have been reported for linewidth narrowing, generally using a frequency discriminator, such as a high-finesse cavity [25] or a molecular resonance [26], [27], to measure the laser frequency fluctuations and generate an error signal that is fed back into the applied current. Other stabilization techniques involve phase locking the quantum cascade laser to a stable frequency reference, such as a frequency comb [2], [28], or compensating frequency fluctuations of the optical power in the QCL by using a laser diode [29].

**Figure 3:**
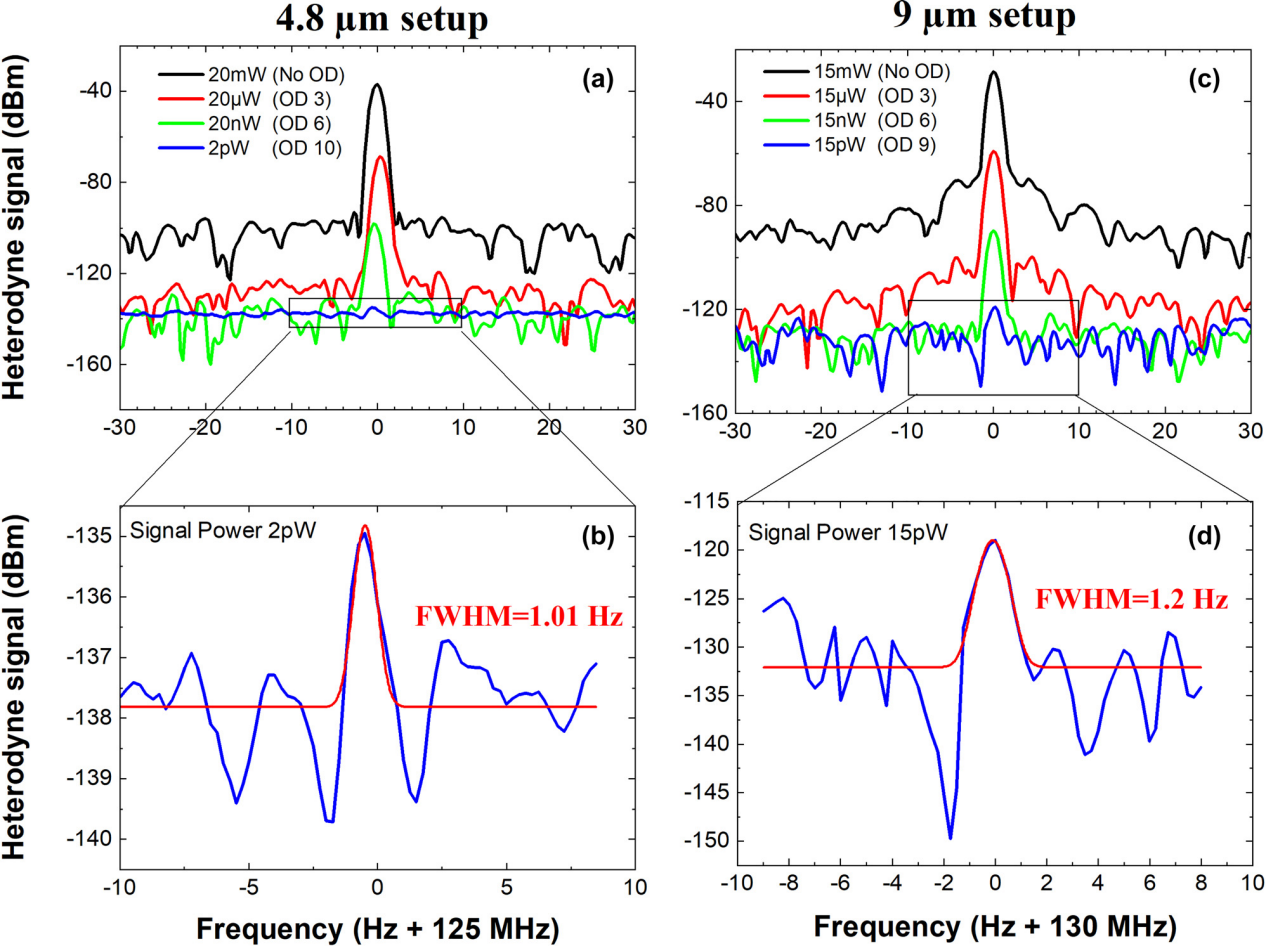
Top panels: beat-note measured for different attenuated signal powers as indicated in the legend at a RBW of 1 Hz (left and right: 4.8 µm and 9 µm setup, respectively). Lower panels: Gaussian fit (red line) of the heterodyne signal (RBW = 1 Hz). OD, optical density. FWHM, full width at half the maximum.

In our experiment, the beatnote between the signal and the local oscillator is stabilised by introducing a stabilization arm in the optical setup, as shown in Figure 1(a), which includes a second detector and a homemade PLL. The beatnote measured on the detector is sent to the PLL and compared to a reference RF signal, and the PLL is used to measure the phase difference between the two signals and actively control the injection current of the signal laser to stabilise the beatnote. It is important to underline that for this experiment no amplification stage has been used, and that all the devices are operated at room temperature. Figure 3 presents the heterodyne signals measured with a RBW of 1 Hz with the two stabilized setups at 4.8 µm (panels (a) and (b)) and 9 µm (panels (c) and (d)). In Figure 3(a) and (c), the different spectra are obtained by progressively attenuating the signal optical power. It is important to underline that the full width at half maximum (FWHM) extracted through a Gaussian fit is very close to the RBW = 1 Hz (Figure 3(b)–(d)).

**Figure 4:**
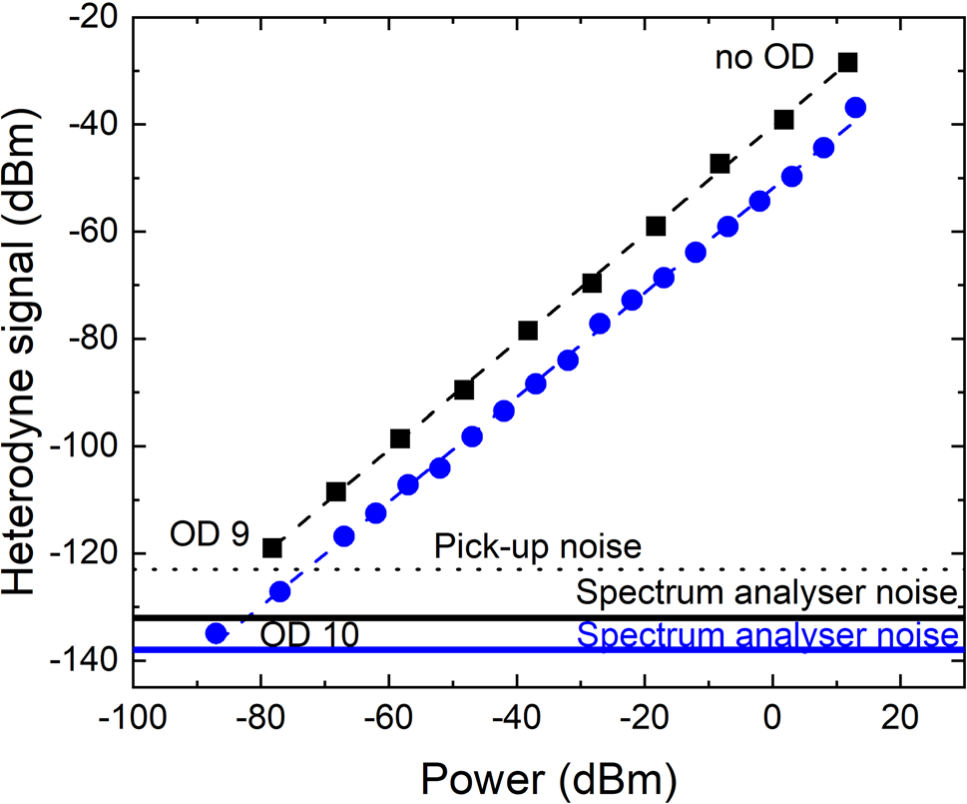
Stabilized setups. Heterodyne power measured at 4.8 µm (blue dots) and 9 µm (black squares) as a function of the optical power of the signal after attenuation. The RBW is 1 Hz and the heterodyne frequency is 125 MHz at 4.8 µm and 130 MHz at 9 µm. Dash-dotted lines are linear fits following Eq. (4). Solid lines indicate the instrument noise floors, while the dotted black line shows the level of electrical pick-up noise in the 9 µm experiment.

The attenuation of the heterodyne peak power for different signal powers is shown in Figure 4. The detected signal now displays a linear dynamic range over more than 9 orders of magnitude, with a slope 0.974 ± 0.008 at 4.8 µm and 1.005 ± 0.008 at 9 µm. When no amplification is present, the heterodyne voltage drops on the load resistance of the instrument

(4) $$P_{\text{het}}=R_{\text{L}}\times I_{\text{het}}^{2}=4R^{2}P_{\text{LO}}\eta^{2}R_{\text{L}}P_{\text{S}}$$

and the values of the heterodyne efficiency extracted from the intercept of the linear fit are 50 % at 4.8 µm and 80 % at 9 µm. Note that, in the 9 µm set-up, the stabilization technique has allowed a more careful alignment of the signal and LO on the detector, resulting in an improved heterodyne efficiency. The lowest power at which the heterodyne signal could be measured is 2 pW (resp. 15 pW) at 4.8 µm (resp. 9 µm), corresponding to 5 × 10^7^ photons per second (resp. 7 × 10^8^ photons per second). Table 3 reports the different noise contributions for a RBW of 1 Hz. The thermal noise has been measured by connecting the detectors to a spectrum analyser (Zurich Instrument UHFLI) after proper amplification to surpass the instrument noise floor, in dark operation. The measured values are systematically higher than those estimated by using Eq. (3). This discrepancy could be attributed to the use of an amplification stage for the measurement. Table 3 shows that in this experiment the spectrum analyser constitutes again the main source of noise. However, we should note that, in the 9 µm experiment, our measurements were affected by pick-up noise (black dotted line in Figure 4), probably due to the packaging of the laser.

**Table 3:** Different noise contributions for 5 µm and 9 µm QCDs at RBW = 1 Hz.

	Thermal noise	Calculated generation – recombination noise	Spectrum analyser noise
4.8 μm QCD	−183.5 dBm (calculated)	−186.4 dBm	−138 dBm
	−176 dBm (measured)
9 μm QCD	−173.5 dBm (calculated)	−183 dBm	−132 dBm
	−168 dBm (measured)

Despite the presence of the pick-up noise at 9 µm, the lowest measured signal power of 15 pW is lower than the NEP extrapolated at 1 Hz in ref. [30].

In the absence of pick-up noise, at 9 µm a NEP of 760 fW (corresponding to 3.4 × 10^7^ photons per second) at 9 µm is expected by intercepting the data curve and the noise floor set by the spectrum analyser. Note that the introduction of an amplifier in the setup did not improve the signal-to-noise ratio of the heterodyne detection, and therefore it was not included in the experiment. Table 4 contains a summary of the values of the NEP obtained with the different techniques, including direct detection. We can see that, remarkably, the heterodyne technique allows a strong improvement of the sensitivity of the detection in both atmospheric windows, as the NEP is reduced by more than 6 orders of magnitude with respect to direct detection.

**Table 4:** Summary of the values of the noise equivalent power (NEP) obtained with direct detection and heterodyne detection, with free running and stabilized QCLs for the two wavelengths.

	Direct detection	Free running QCLs and amplification	Stabilised beatnote (no amplification)
NEP @ 4.8 µm (W)	3.7 × 10^−6^	9.6 × 10^−10^	1.5 × 10^−12^
NEP @ 9 µm (W)	1.3 × 10^−6^	2 × 10^−10^	6 × 10^−12^ (7.6 × 10^−13^ without pick-up noise)

As the heterodyne frequency used in this experiment is lower than the high frequency cut-off of a typical Peltier cooled (operating at 200 K) commercial mercury cadmium telluride (MCT) detector, one could ask how our room temperature QCD and MCTs compare as heterodyne receivers (see Supplementary Information). We estimate that, considering the same heterodyne efficiency as in our experiment, the NEP expected from a heterodyne set-up employing a commercial MCT detector could be 40 dBm lower than the one we measured. This difference could be easily compensated by pre-amplifying the QCD, as the noise level is not limited by the device (see Table 3). When using the QCD as heterodyne receiver, the NEP could be further reduced if a waveguide [31] or metamaterial [32] architecture is employed for the device. Furthermore, the local oscillator power can be increased up to a few hundreds of mW when using the QCD, while MCTs usually saturate for an impinging power of few hundreds µW. QCDs have, therefore, the full potential to be high sensitivity heterodyne receivers operating room temperature and with frequency response of few tens of GHz [1].

## 5 Conclusions

In conclusion, we exploited the peculiar characteristics of unipolar quantum devices combined with an active stabilization technique, to demonstrate heterodyne detection in the mid-infrared atmospheric windows with pW sensitivity. The detectors operate at room temperature, without any amplification stage and show a linear behaviour for more than 9 orders of magnitude.

In order to estimate the maximum achievable sensitivity of the heterodyne setup, we consider the limit where the most important contribution to the noise comes from the generation – recombination noise, and where the signal power is negligible with respect to that of the local oscillator. In this limit, and considering unity heterodyne efficiency, the signal-to-noise ratio can be written as:

$$\frac{S}{N}=\frac{2R\sqrt{P_{\text{LO}}P_{\text{S}}}}{\sqrt{4eg\Delta fRP_{\text{LO}}}}=\sqrt{\frac{\alpha P_{\text{s}}}{E\Delta f}}$$

where the responsivity has been expressed in terms of the absorption quantum efficiency, *α*, and the photon energy, *E*, as: R=eαg/E . The minimum achievable NEP is thus:

(5) $$\text{NEP}=\frac{E\Delta f}{\alpha }$$

At 9 µm wavelength, the NEP associated with the detection of one photon per second corresponds to 0.02 aW. Equation (5) shows that the heterodyne signal is ultimately limited by the absorption quantum efficiency of the detector, and by the resolution bandwidth. In this work, we have been focusing on the reduction of the bandwidth through the active stabilisation of the lasers. An interesting perspective to improve the absorption quantum efficiency concerns the use of photodetectors based on patch antenna resonators [15], [30]. They have already been successfully implemented in heterodyne detection setups and can be packaged in high speed architectures [17] and designed to operate in the critical coupling regime [31]. Furthermore, they operate in reflectivity rather than in transmission, which could be an advantage for optical alignment.

## Supplementary Material

Supplementary Material Details
