# Supplementary material for: Ultra-sensitive heterodyne detection at room temperature in the atmospheric windows
Source: Nanophotonics. 2024 Jan 18;13(10):1765–72. doi: 10.1515/nanoph-2023-0787 (PMC11501748; doi:10.1515/nanoph-2023-0787)
Supplement: Supplementary file 1 — Supplementary Material Details [file j_nanoph-2023-0787_suppl_001.pdf]

Ultra-sensitive heterodyne detection at room temperature in the atmospheric windows

Supporting information

Mohammadreza Saemian[1,*], Livia Del Balzo[1,*], Djamal Gacemi[1], Yanko Todorov[1], Etienne Rodriguez[1], Olivier Lopez[2], Benoit Darquié[2], Lianhe Li[3], Alexander Giles Davies[3], Edmund Linfield[3], Angela Vasanelli[1,#], Carlo Sirtori[1]

[1]Laboratoire de Physique de l'Ecole Normale Supérieure, ENS, Université PSL, CNRS, Sorbonne Université, Université Paris Cité, 75005 Paris, France

[2]Laboratoire de Physique des Lasers, CNRS, Université Sorbonne Paris Nord, 93430 Villetaneuse, France

[3]School of Electronics and Electrical Engineering, University of Leeds, Woodhouse Lane, Leeds LS2 9JT, UK

*These authors contributed equally to this work.
#Corresponding author: angela.vasanelli@ens.fr

1. Description of the quantum cascade detectors

The quantum cascade detector (QCD) used in the 4.8 µm set-up, centered at 5 µm, is composed of 12 periods of lattice matched InGaAs wells and InAlAs barriers, grown on an InP substrate by Molecular Beam Epitaxy. In a single period, the thicknesses, in nm, of the InGaAs wells and AlInAs barriers (indicated in bold) are the following: <u>3.8</u>/**1.8**/0.9/**6.2**/1.3/**5.5**/1.7/**5.0**/2.4/**3.8**, with the first underlined layer being an InGaAs quantum well, doped at $3 \times 10^{18}\ \mathrm{cm}^{-3}$. The conduction band diagram with the square moduli of the relevant wavefunctions, plotted at the corresponding energies, is shown in Figure 1(b) in the main text.

The 9 µm QCD structure is composed of 12 periods of GaAs wells and $Al_{0.35}Ga_{0.65}As$ barriers grown by Molecular Beam epitaxy on a GaAs substrate. The single period structure is (all the thicknesses are in nm): <u>4.4</u>/**1.4**/1.4/**5.5**/1.7/**5.8**/2.3/**5.2**/3.0/**4.8** with the first underlined layer being GaAs well doped at $1 \times 10^{18}\ \mathrm{cm}^{-3}$, while the barrier thicknesses are indicated in bold. The conduction band diagram with the square moduli of the relevant wavefunctions, plotted at the corresponding energies, is shown in Figure S1.

The QCDs were processed into high-frequency coupled mesas[1,2,3]. Square mesa regions of 50 µm size were defined through optical lithography. In the case of the 5 µm QCD, the mesas were chemically etched down to the bottom contact layer using H_3PO_4:H_2O_2:H_2O (1:1:38). Then the half plane starting just below the mesa was protected with resist, allowing the contact to be etched away (using the same H_3PO_4 solution) down to the substrate. After depositing a sacrificial support of reflowed S1818 resist for the air bridge, a Ti/Au 50 Ω coplanar waveguide was evaporated in one step using negative resist AZ5214 E for patterning. A photoresist stripper (SVC 14) was preferred to the standard acetone lift off to ensure that the bridge was properly freed from resist.

In the case of the 9 µm QCD, physical etching instead of wet chemical etching, was used, as explained in ref. [1].

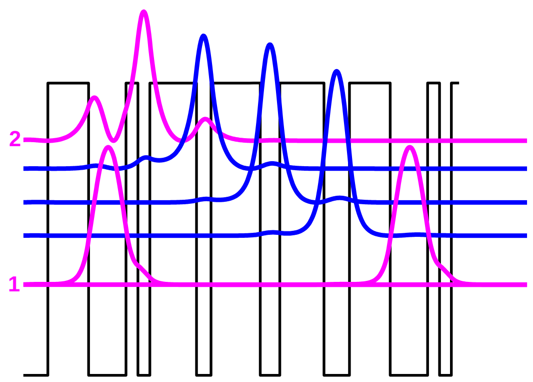

Figure S1 : Band structure of the 9 µm QCD. The square moduli of the wavefunctions involved in the photon absorption are indicated in purple, while those involved in the electron extraction are indicated in blue.

2. QCD responsivity

To measure the responsivity of the devices, a DFB QCL (λ=4.8 µm from Ad Tech Optics and λ=9 µm from Thorlabs) is focused on the active area of the QCD with a F=12.5 mm lens. The generated photocurrent is read on a sourcemeter (Keithley 2450), while the incident power is measured with a powermeter by means of a flipping mirror that can deviate the optical path (experimental setup in Figure S2).

Figure S3 shows the photocurrent of the two devices measured at room temperature as a function of the QCL power impinging on the detector. The responsivity extracted from the slope is 1.9 mA/W for the 5 µm device and 4.2 mA/W for the 9 µm device.

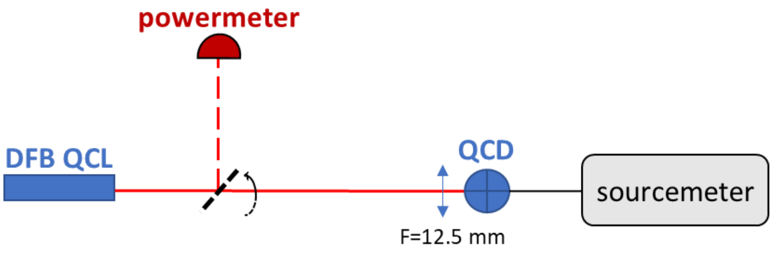

Figure S2. Sketch of the setup for responsivity measurements.

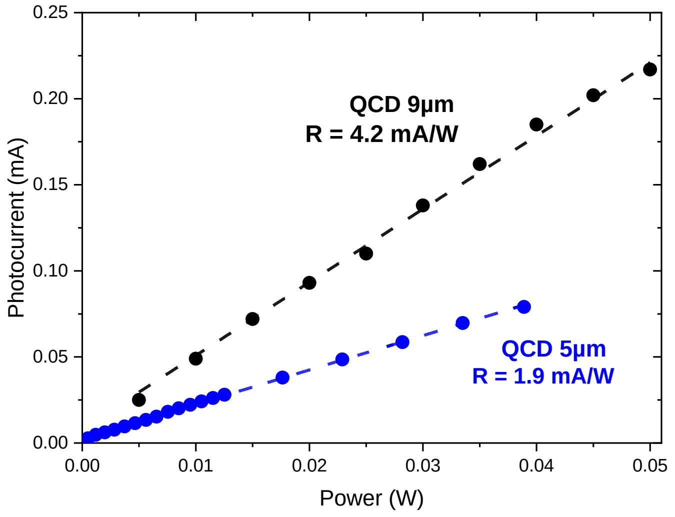

Figure S3. QCDs photocurrent vs laser power. Dashed lines show the linear fit of the data, from which the responsivity is extracted.

3. QCDs frequency response

The frequency response of the QCDs was measured by using the rectification technique, that relies on the non-linear current-voltage (I-V) characteristic of the detector [4]. The experimental setup is shown in Figure S4. A sinusoidal radiofrequency signal generated by a synthesizer (Anritsu MG3693B) is sent to the QCD through the AC port of a bias-tee. The rectified current, extracted through the DC port of the bias-tee, is measured on a sourcemeter (Keithley 2450). The rectified current, which is proportional to the squared voltage transfer function, $H(\omega)$ of the device, is collected while sweeping the frequency of the AC signal up to 30 GHz. Figure S5 shows the rectified power (i.e. the squared rectified current times the load resistance) as a function of the AC frequency for the 5 µm detector. A 3dB cutoff frequency of 2 GHz is extracted from this plot. Similar measurements were performed for the 9 µm QCD, and the corresponding results have been presented in ref. [1]. The measured cutoff frequency for the 9 µm detector was 5 GHz.

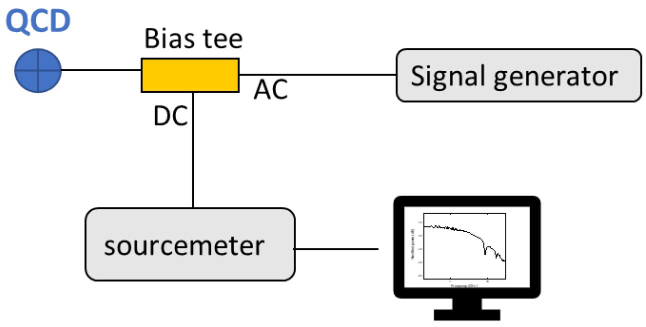

Figure S4: Experimental setup for rectification measurements.

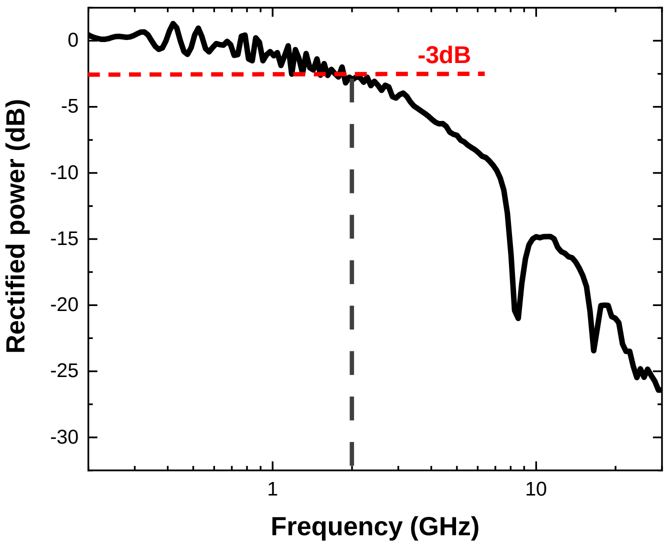

Figure S5. Rectified power plotted as a function of the AC frequency for the 5 µm QCD.

4. Estimated NEP of the QCDs in direct detection

The Noise Equivalent Power (NEP) of the QCDs in direct detection is:[5]

$$NEP = \frac{i_n}{\mathcal{R}}$$

where i_n is the current noise and $\mathcal{R}$ responsivity of the detector (see section 2). In direct detection, the measurement bandwidth is limited by the QCL linewidth, which is 1 MHz at 4.8 µm and 100 kHz at 9 µm. The most important contribution to the noise is the thermal noise, which is estimated to be -116 dBm for the 4.8 µm QCD and -118 dBm for the 9 µm QCD. This estimation is performed starting from the measured thermal noise power at 1Hz (see main text), and considering the measurement bandwidth. From these values it is possible to deduce the current noise as:

$$i_n = \sqrt{\frac{P_{thermal\,noise}\,(Watt)}{50\,\Omega}}$$

We finally obtain a NEP of 3.7 µW at 4.8 µm and 1.34 µW 9 µm.

	Measured thermal noise power at 1Hz	Thermal noise intensity in direct detection	Thermal current noise i_n	NEP in direct detection
4.8 µm QCD	-176 dBm	-116 dBm @1MHz	7.1 nA	3.7 µW
9 µm QCD	-168 dBm	-118 dBm @100kHz	5.6 nA	1.34 µW

Table S1. Estimation of the NEP of the QCD detectors. The thermal noise is measured with a measurement bandwidth of 1Hz (see main text). The thermal noise intensity is then estimated by considering the measurement bandwidth set by the linewidth of the QCLs. The thermal current noise and then the NEP are finally extracted from this value.

5. Comparison with a commercial Peltier cooled Mercury Cadmium Telluride detector

In this section we compare the capabilities of our QCD as heterodyne receiver at 9 µm with respect to a commercial Peltier cooled Mercury Cadmium Telluride (MCT) detector (Vigo PVI-4TE-10.6-0.5x0.5).

The characteristics of the two devices that are relevant for heterodyne detection are summarized in Table S2.

	Operation temperature	High cut-off frequency	Responsivity	Pre-amplification	Noise level @ 1Hz	Saturation power
Peltier cooled MCT	200 K	700 MHz	2.55E+4 V/W	Yes (8.65E+3 V/A)	-120 dBm	200 µW
QCD	300 K	3 GHz	4 mA/W	Non	-168 dBm	> 100 mW

Table S2. Comparison of the measured characteristics of a commercial Peltier cooled MCT detector and of our 9 µm QCD.

The QCD is a photovoltaic device that does not need to be cooled. As it operates at 0 V and it is not amplified, its noise level is typically lower than the MCT's one. At room temperature the QCD responsivity is of few mA/W, three orders of magnitude lower than the MCT's one. One should note that, contrarily to the QCD, the MCT is pre-amplified, with a transimpedance gain of 10^4 V/A. The responsivity of QCD can be increased by using metamaterial - based devices (50 mA/W at 0V and room temperature in ref. [6]) or waveguide geometries (400 mA/W in ref. [7]). The QCD can also be pre-amplified, as we did in the experiment presented in section 3 of the main manuscript.

An important property for heterodyne detection is the saturation power of the detector. For the MCT detector, the saturation power is 200 µW. In the case of the QCD, we measured a linear behaviour of the photocurrent as a function of the incident QCL power up to 100 mW (the maximum power delivered by the QCL)-. However, the theoretical saturation intensity of the QCD could be much higher, as in an intersubband system at ~10 µm it can reach 1MW/cm².[8] This means that a local oscillator with a power up to three orders of magnitude higher than that used in our experiment could be employed if an intersubband detector is used as local oscillator.

Finally, an important difference between the two devices is their frequency bandwidth. Figure S6 presents a direct optical measurement of the frequency bandwidth obtained by shining a mid-infrared frequency comb (Menlo System FC1500-ULN) onto the detector. The beating between the optical teeth appears as beatnotes separated by 100 MHz. From this figure, we extract a -3dB cut-off of 3 GHz for the 9 µm QCD, and of 700 MHz for the MCT detector.

In the experiment discussed in the main text, the heterodyne signal is at 130 MHz, limited by the PLL cut-off frequency. As this frequency is lower than the high frequency cut-off of the MCT detector, one could ask whether a commercial Peltier cooled MCT could provide a lower NEP than the QCD in a heterodyne detection experiment. We estimate that, assuming the same heterodyne efficiency as in our experiment , the NEP expected from a heterodyne set-up employing the commercial detector presented above could be 40 dBm lower than the one we measured. This difference could be easily compensated by pre-amplifying the detector, as the noise level is not limited by the device.

Several improvements can be introduced in the QCD architecture and packaging in order to reduce the NEP. Indeed, one can use a QCD based on a waveguide geometry, which can be also pre-amplified and cooled by using a packaging similar to the MCT's one. Furthermore, the local oscillator power can be increased up to a few hundreds of mW.

Considering a QCD detector with 0.5A/W responsivity (which is feasible if considering a Peltier cooled device in a waveguide geometry) and a local oscillator power of 200 mW, we estimate a noise equivalent power of 10 aW, corresponding to the detection of 500 photons per second.

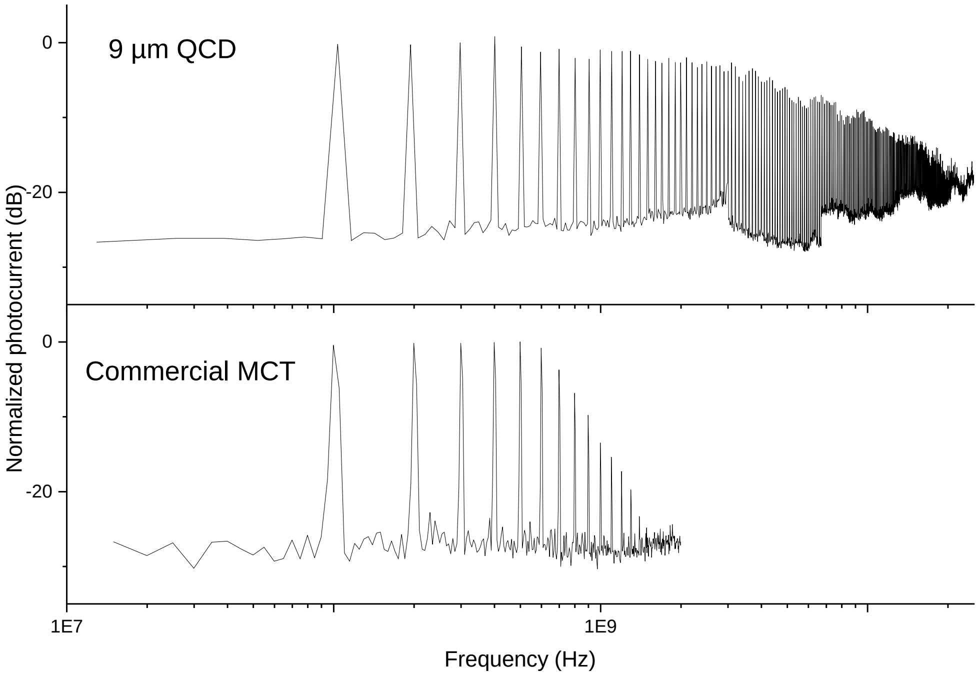

Figure S6. Normalized photocurrent measured on the 9 µm QCD and on the commercial MCT by using a mid-infrared frequency comb with teeth separated of 100 MHz.

References

[1] H. Dely, T. Bonazzi, O. Spitz, E. Rodriguez, D. Gacemi, Y. Todorov, K. Pantzas, G. Beaudoin, I. Sagnes, L. Li, A.G. Davies, E.H. Linfield, F. Grillot, A. Vasanelli, and C. Sirtori, "10 Gbit s $^{-1}$ Free Space Data Transmission at 9 µm Wavelength With Unipolar Quantum Optoelectronics," Laser & Photonics Reviews **16**(2), 2100414 (2022).

[2] E. Rodriguez, A. Mottaghizadeh, D. Gacemi, D. Palaferri, Z. Asghari, M. Jeannin, A. Vasanelli, A. Bigioli, Y. Todorov, M. Beck, J. Faist, Q.J. Wang, and C. Sirtori, "Room-Temperature, Wide-Band, Quantum Well Infrared Photodetector for Microwave Optical Links at 4.9 µm Wavelength," ACS Photonics **5**(9), 3689–3694 (2018).

[3] P.D. Grant, R. Dudek, M. Buchanan, and H.C. Liu, "Room-Temperature Heterodyne Detection up to 110 GHz With a Quantum-Well Infrared Photodetector," IEEE Photon. Technol. Lett. **18**(21), 2218–2220 (2006).

[4] H.C. Liu, J. Li, M. Buchanan, and Z.R. Wasilewski, "High-frequency quantum-well infrared photodetectors measured by microwave-rectification technique," IEEE Journal of Quantum Electronics **32**(6), 1024–1028 (1996).
[5] E. Rosencher, and B. Vinter, *Optoelectronics*, 1st ed. (Cambridge University Press, 2002).
[6] A. Bigioli, G. Armaroli, A. Vasanelli, D. Gacemi, Y. Todorov, D. Palaferri, L. Li, A.G. Davies, E.H. Linfield, and C. Sirtori, "Long-wavelength infrared photovoltaic heterodyne receivers using patch-antenna quantum cascade detectors," Applied Physics Letters **116**(16), 161101 (2020).
[7] G. Marschick, M. David, E. Arigliani, N. Opačak, B. Schwarz, M. Giparakis, A. Delga, M. Lagree, T. Poletti, V. Trinite, A. Evirgen, B. Gerard, G. Ramer, R. Maulini, J. Butet, S. Blaser, A.M. Andrews, G. Strasser, and B. Hinkov, "High-responsivity operation of quantum cascade detectors at 9 µm," Opt. Express **30**(22), 40188 (2022).
[8] M. Jeannin, J.-M. Manceau, and R. Colombelli, "Unified Description of Saturation and Bistability of Intersubband Transitions in the Weak and Strong Light-Matter Coupling Regimes," Phys. Rev. Lett. **127**(18), 187401 (2021).
